# A Review of Bayesian Hypothesis Testing and Its Practical Implementations

**DOI:** 10.3390/e24020161

**Published:** 2022-01-21

**Authors:** Zhengxiao Wei, Aijun Yang, Leno Rocha, Michelle F. Miranda, Farouk S. Nathoo

**Affiliations:** Department of Mathematics and Statistics, University of Victoria, Victoria, BC V8W 2Y2, Canada; zhengxiao@uvic.ca (Z.W.); aijunyan@uvic.ca (A.Y.); lenorocha@uvic.ca (L.R.); michellemiranda@uvic.ca (M.F.M.)

**Keywords:** hypothesis testing, Bayes factor, prior distributions

## Abstract

We discuss hypothesis testing and compare different theories in light of observed or experimental data as fundamental endeavors in the sciences. Issues associated with the *p*-value approach and null hypothesis significance testing are reviewed, and the Bayesian alternative based on the Bayes factor is introduced, along with a review of computational methods and sensitivity related to prior distributions. We demonstrate how Bayesian testing can be practically implemented in several examples, such as the *t*-test, two-sample comparisons, linear mixed models, and Poisson mixed models by using existing software. Caveats and potential problems associated with Bayesian testing are also discussed. We aim to inform researchers in the many fields where Bayesian testing is not in common use of a well-developed alternative to null hypothesis significance testing and to demonstrate its standard implementation.

## 1. Introduction

Hypothesis testing is an important tool in modern research. It is applied in a wide range of fields, from forensic analysis, business intelligence, and manufacturing quality control, to the theoretical framework of assessing the plausibility of theories in physics, psychology, and fundamental science [1,2,3,4,5]. The task of comparing competing theories based on data is essential to scientific activity, and therefore, the mechanism of conducting these comparisons requires thoughtful consideration [6,7].

The dominant approach for these comparisons is based on hypothesis testing using a *p*-value, which is the probability, under repeated sampling, of obtaining a test statistic at least as extreme as the observed under the null hypothesis [4,8]. Records of conceptualizing the *p*-value date back at least two hundred years before Ronald Fisher established the *p*-value terminology and technique [9,10,11]. These records are an indication of how compelling and popular the approach is, and the long history explains the widespread acceptance of a decision rule with a fixed type I error rate, which further resulted in the adoption of a 5% significance-level cutoff. Despite its prevalence, there has been an intense debate about the misuse of the *p*-value approach [7,12]. The major criticisms about the *p*-value are its inability to quantify evidence for the null hypothesis and its tendency to overestimate the evidence against the null hypothesis [4]. For example, a possible decision based on the *p*-value is the rejection of the null hypothesis but not its acceptance. Under the null hypothesis, the *p*-value will have a uniform [0, 1] distribution regardless of the sample size. This is by construction. The Bayesian approach behaves rather differently under the null hypothesis, and increasing sample sizes will provide increasing degrees of evidence in favor of the null hypothesis [13].

Besides the misuse, the hypothesis testing approach based on the *p*-value can be easily misinterpreted. A list of twenty-five examples of misinterpretations in classical hypothesis testing is provided in [14]. Eighteen of these items are directly related to the misunderstanding of the *p*-value, and the others are related to *p*-values in the context of confidence intervals and statistical power. Some of these points are also shared in [15], including the common misconceptions that a nonsignificant difference means that there is no difference between groups and that the *p*-value represents the chance of a type I error. The author also highlights an alternative approach, based on the Bayes factor as a measure of true evidential meaning about the hypotheses [16,17]. Private pages of Alan Turing independently discovered this quantity around the same time as Jeffrey [16,18,19]. Other authors have also recommended the Bayes factor as a better solution to hypothesis testing compared with the practice of *p*-values and null hypothesis significance testing (NHST), specifically criticizing the *p*-value’s dependence on hypothetical data, which are likely to be manipulated by the researcher’s intentions [8].

While the majority of the issues with classical hypothesis testing are crucial and widely known, a less acknowledged but important misinterpretation happens when two or more results are compared by their degrees of statistical significance [20]. To illustrate this issue, consider the following example introduced in [14]. Suppose two independent studies have effect estimates and standard errors of 25±10 and 10±10. In that case, the first study has a mean that is 2.5 standard errors away from 0, being statistically significant at an alpha level of 1%. The second study has a mean that is 1 standard error away from 0 and is not statistically significant at the same alpha level. It is tempting to conclude that the results of the studies are very different. However, the estimated difference in treatment effects is 25−10=15, with a standard error $\sqrt{10^{2}+10^{2}}\approx 14$. Thus, the mean of 15 units is less than 1 standard error away from 0, indicating that the difference between the studies is not statistically significant. If a third independent study with a much larger sample size had an effect estimate of 2.5±1.0, then it would have a mean that is 2.5 standard errors away from 0 and indicate statistical significance at an alpha level of 1%, as in the first study. In this case, the difference between the results of the third and the first studies would be 22.5 with a standard error $\sqrt{10^{2}+1}\approx 10$. Thus, the mean of 22.5 units would be more than 2 standard errors away from 0, indicating a statistically significant difference between the studies. Therefore, the researchers in [20] recommend that the statistical significance of the difference between means be considered, rather than the difference between the significance levels of the two hypotheses.

To prevent the misuse and misinterpretation of *p*-values, the American Statistical Association (ASA) issued a statement clarifying six principles for the proper use and interpretation of classical significance testing [12]: (i) *p*-values can indicate how incompatible the data are with a specified statistical model; (ii) *p*-values do not measure the probability that the studied hypothesis is true, or the probability that the data were produced by random chance alone; (iii) scientific conclusions and business or policy decisions should not be based only on whether a *p*-value passes a specific threshold; (iv) proper inference requires full reporting and transparency; (v) *p*-value, or statistical significance, does not measure the size of an effect or the importance of a result; and (vi) by itself, a *p*-value does not provide a good measure of evidence regarding a model or hypothesis.

The profound criticism of the *p*-value approach has promoted the consideration and development of alternative methods for hypothesis testing [4,8,12,21]. The Bayes factor is one such instance [18,22], since it only depends on the observed data and allows an evaluation of the evidence in favor of the null hypothesis. The seminal paper by Kass and Raftery [17] discusses the Bayes factor along with technical and computational aspects and presents several applications in which the Bayes factor can solve problems that cannot be addressed by the *p*-value approach. Our review differs in that it is targeted towards researchers in fields where the *p*-value is still in dominant use, and there are many such fields where this is the case. Our emphasis is to provide these researchers with an understanding of the methodology and potential issues, and a review of the existing tools to implement the Bayes factor in statistical practice.

Two potential issues for the implementation of the Bayes factor are the computation of integrals related to the marginal probabilities that are required to evaluate them and the subjectivity regarding the choosing of the prior distributions [7,17]. We will review these issues in Section 2 and Section 3, respectively. Despite these difficulties, there are many advantages to the use of the Bayes factor, including (i) the quantification of the evidence in favor of the null hypothesis [15], (ii) the ease of combining Bayes factors across experiments, (iii) the possibility of updating results when new data are available, (iv) interpretable model comparisons, and (v) the availability of open-source tools to compute Bayes factors in a variety of practical applications.

This paper aims to provide examples of practical implementations of the Bayes factor in different scenarios, highlighting the availability of tools for its computation for those with a basic understanding of statistics. In addition, we bring attention to the over-reliance on the classical *p*-value approach for hypothesis testing and its inherent pitfalls. The remainder of the article is structured as follows. In Section 2, we define the Bayes factor and discuss technical aspects, including its numerical computation. In Section 3, we discuss prior distributions and the sensitivity of the Bayes factor to prior distributions. Section 4 presents several applications of the Bayes factor using open-source code involving R software. We illustrate the computation of the Bayes factor using a variety of approximation techniques. Section 5 presents a discussion and summary.

## 2. Bayes Factor Definition and Technical Aspects

### 2.1. Definition

The Bayes factor is defined as the ratio of the probability of the *observed data*, conditional on two competing hypotheses or models. Given the same data *D* and two hypotheses $H_{0}$ and $H_{1}$, it is defined as

(1) $$BF_{10}=\frac{p(D|H_{1})}{p(D|H_{0})}.$$

If there is no previous knowledge in favor of one theory over the other, i.e., the hypotheses $H_{0}$ and $H_{1}$ are equally probable a priori ($p\left(H_{1}\right)=p\left(H_{0}\right)$), the Bayes factor represents the ratio of the data-updated knowledge about the hypotheses, i.e., the Bayes factor is equal to the posterior odds, where the posterior probability is defined as the conditional probability of the hypothesis given the data. Using the definition of conditional probability and under the assumption that the hypotheses are equally probable a priori,

(2) $$BF_{10}=\frac{p(D|H_{1})}{p(D|H_{0})}=\frac{p\left(H_{1}|D\right)/p\left(H_{1}\right)}{p\left(H_{0}|D\right)/p\left(H_{0}\right)}=\frac{p(H_{1}|D)}{p(H_{0}|D)}.$$

Based on Equation (2), we can interpret the Bayes factor as the extent to which the data update the prior odds, and therefore, quantify the support for one model over another. A Bayes factor value smaller than one indicates that the data are more likely under the denominator model than they are under the numerator model. A model with the highest Bayes factor shows the relatively highest amount of evidence in favor of the model compared to the other models. Similarly, by switching the indices in (1), $BF_{01}$ is defined as

(3) $$BF_{01}=\frac{p(D|H_{0})}{p(D|H_{1})},$$

where larger values of $BF_{01}$ represent higher evidence in favor of the null hypothesis.

The Bayes factor can be viewed as a summary of the evidence given by data in support of one hypothesis in contrast to another [7,17]. Reporting Bayes factors can be guided by setting customized thresholds according to particular applications. For example, Evett [1] argued that for forensic evidence alone to be conclusive in a criminal trial, it would require a Bayes factor of at least 1000 rather than the value of 100 suggested by the Jeffreys scale of interpretation [18]. A generally accepted table provided in [17] is replicated in Table 1, and other similar tables are available in [21]. Thus, using the Bayes factor can result in reporting evidence in favor of the alternative hypothesis, evidence in favor of the null hypothesis, or reporting that the data are inconclusive.

The Bayes factor can avoid the drawbacks associated with *p*-values and assess the strength of evidence in favor of the null model along with various additional advantages. First, Bayes factors inherently include a penalty for complex models to prevent overfitting. Such a penalty is implicit in the integration over parameters required to obtain marginal likelihoods. Second, the Bayes factor can be applied in statistical settings that do not satisfy common regularity conditions [17].

Despite its apparent advantages, there are a few disadvantages to the Bayes factor approach. First, the choice of a prior distribution is subjective [4,7,17] and might be a concern for some researchers. However, the authors in [7] challenge the criticism, claiming that there is nothing about the data, by itself, that assures it counts as evidence. The pathway from the data to evidence is filled with subjective evaluations when combing the theoretical viewpoint with the research question. Therefore, the Bayesian approach makes explicit assumptions based on the prior likelihood statement. A way to avoid the explicit selection of prior densities is through the use of the Bayesian information criterion (BIC), which can give a rough interpretation of evidence in Table 1.

Another potential disadvantage is the computational difficulty of evaluating marginal likelihoods, and this is discussed in Section 2.2. However, the issue is being mitigated by the growth of computational power and the availability of open-source statistical tools for this computation. Examples of these tools are *BayesFactor*, *brms*, and *BFpack* R packages [23,24,25]; and JASP [26] software. In Section 4, we illustrate the required R scripting for a number of examples widely used in data analysis. As Python has become increasingly popular among quantitative practitioners [27,28], R packages for the computation of Bayes factors can be imported into Python using the *rpy2* package [29]. Thanks to these advancements, Bayes factors are gradually gaining wider attention in research [30,31,32].

### 2.2. Computation of the Bayes Factor

To calculate the Bayes factor, both the numerator and the denominator in the Bayes factor definition (1) (the marginal likelihood of the data under a given model) involve integrals over the parameter space:

(4) $$p\left(D|H_{k}\right)=\int_{\Theta_{k}}p\left(D|\theta_{k}\right)p\left(\theta_{k}|H_{k}\right)d\theta_{k},$$

where $\theta_{k}$ is the parameter vector under the hypothesis $H_{k}$, and $p(\theta_{k}|H_{k})$ is the prior probability density function of the parameter vector for the hypothesis $H_{k}$. It is typical for (4) to be an integral over many dimensions so that the computational problem can be difficult.

If we assume the data are a random sample from an exponential family distribution and assume conjugate priors, it is possible to solve the integral in (4) analytically. Without conjugacy, these integrals are often intractable, and numerical methods are needed. Many available numerical integration techniques are inefficient to calculate such integrals because it is difficult to find the regions where the probability mass is accumulating in higher dimensions. For regular problems in the large sample setting, the probability mass will accumulate and tend to peak around the maximum likelihood estimator (MLE) [17,33]. This notion underlies the Laplace approximation and its variations which can be used to obtain an approximation to the Bayes factor. These methods rely on a quadratic approximation to the logarithm of the integrand obtained using a Taylor expansion about the MLE and a normal distribution matching. Laplace’s methods are usually fast but not very accurate. An alternative approximation known as the *Savage–Dickey density ratio* [34] can be applied to obtain a better approximation for the case of nested models when testing a constrained model against an unrestricted alternative, the Bayes factor is approximated by dividing the value of the posterior density over the parameters for the alternative model evaluated at the hypothesized value, by the prior for the same model evaluated at the same point [35].

For the general case of Bayes factor computations, it is common to resort to sampling-based numerical procedures adjusted to the context of marginal likelihood computation as in (4). Evans and Swartz [36] reviewed several numerical strategies for assessing the integral related to the Bayes factor and later published a book on the topic [37]. Among the methods for estimating the integral of the marginal likelihood, the bridge sampling technique has gained prominence [38]. The method encompasses three special cases, namely the “naïve” [33] or “simple” [17] Monte Carlo estimator, the importance sampling, and the generalized harmonic mean estimator. The bridge sampling estimate stands out for not being dominated by samples from the tails of the distribution [33]. An entitled *bridgesampling* R package to estimate integrals with the bridge sampling algorithm for Bayesian models implemented in Stan [39] or JAGS [40] is available [41]. In Section 4, we provide examples of using the *bridgesampling* package and the *BayesFactor* R package [23] to enable the computation of Bayes factors for several important experimental designs.

## 3. Prior Elicitation and Sensitivity Analysis

Based on its definition in (1), the Bayes factor is a ratio of the marginal likelihood of two competing models. The marginal likelihood for a model class is a weighted average of the likelihood over all the parameter values represented by the prior distribution [42]. Therefore, carefully choosing priors and conducting a prior sensitivity analysis play an essential role when using Bayes factors as a model selection tool. This section briefly discusses the prior distributions, prior elicitation, and prior sensitivity analysis.

### 3.1. Prior Distributions

In Bayesian statistical inference, a prior probability distribution (or simply called the prior) estimates the probability of incorporating one’s beliefs or prior knowledge about an uncertain quantity before collecting the data. The unknown quantity may be a parameter of the model or a latent variable. In Bayesian hierarchical models, we have more than one level of prior distribution corresponding to a hierarchical model structure. The parameters of a prior distribution are called hyperparameters. We can either assume values for the hyperparameters or assume a probability distribution, which is referred to as a hyperprior.

It is common to categorize priors into four types: informative priors, weakly informative priors, uninformative priors, and improper priors [43]. The Bayes factor computation requires proper priors, i.e., a prior distribution that integrates to 1. Various available software provide default priors, but it is the researchers’ responsibility to perform sensitivity analysis to check the impact of applying different priors.

### 3.2. Prior Elicitation

The prior distribution is an important ingredient of the Bayesian paradigm and must be designed coherently to make Bayesian inference operational [44]. Priors can be elicited using multiple methods, e.g., from past information, such as previous experiments, or elicited purely from the experts’ subjective assessments. When no prior information is available, an uninformative prior can be assumed, and most of the model information that is given by the posterior will come from the likelihood function itself. Priors can also be chosen according to some principles, such as symmetry or maximum entropy, given constraints. Examples are the Jeffreys prior [18] and Bernardo’s reference prior [45]. When a family of conjugate priors exist, choosing a prior from that family simplifies the calculation of the posterior distribution.

With the advancement of computational power, ad hoc searching for priors can be done more systemically. Hartmann et al. [46] utilized the prior predictive distribution implied by the model to automatically transform experts’ judgments about plausible outcome values to suitable priors on the parameters. They also provided computational strategies to perform inference and guidelines to facilitate practical use. Their methodology can be summarized as follows: (i) define the parametric model for observable data conditional on the parameters θ and a prior distribution with hyperparameters λ for the parameters θ, (ii) obtain experts’ beliefs or probability for each mutually exclusive data category partitioned from the overall data space, (iii) model the elicited probabilities from step 2 as a function of the hyperparameters λ, (iv) perform iterative optimization of the model from step 3 to obtain an estimate for λ best describing the expert opinion within the chosen parametric family of prior distributions, and (v) evaluate how well the predictions obtained from the optimal prior distribution can describe the elicited expert opinion. Prior predictive tools relying on machine learning methods can be useful when dealing with hierarchical modeling where a grid search method is not possible [47].

### 3.3. Sensitivity Analysis

In the Bayesian approach, it is important to evaluate the impact of prior assumptions. This is performed through a sensitivity analysis where the prior is perturbed, and the change in the results is examined. Various authors have demonstrated how priors affect Bayes factors and provided ways to address the issue. When comparing two nested models in a low dimensional parameter space, the authors in [48] propose a point mass prior Bayes factor approach. The point mass prior distribution for the Bayes factor is computed for a grid of extra parameter values introduced by a generalized alternative model. The resulting Bayes factor is obtained by averaging the point mass prior Bayes factor over the prior distribution of the extra parameter(s).

For binomial data, Ref. [42] shows the impact of different priors on the probability of success. The authors used four different priors: (i) a uniform distribution, (ii) the Jeffreys prior, which is a proper Beta(0.5,0.5) distribution, (iii) the Haldane prior by assuming a Beta(0,0) distribution (an improper prior), and (iv) an informative prior. The uniform, Jeffreys, and Haldane priors are noninformative in some sense. Although the resulting parameter estimation is similar in all four scenarios, the resulting Bayes factor and posterior probability of $H_{1}$ vary. Using the four different priors produces very different Bayes factors with values of 0.09 for the Haldane, 0.6 for the Jeffreys, 0.91 for the uniform, and 1.55 for the informative prior. The corresponding posterior probabilities of $H_{1}$ are 0.08 (Haldane), 0.38 (Jeffreys), 0.48 (uniform), and 0.61 (informative). In this example, the sensitivity analysis reveals that the effect of the priors on the posterior distribution is different from their effect on the Bayes factor. The authors emphasize that Bayes factors should be calculated, ideally, for a wide range of plausible priors whenever used as a model selection tool. Besides using the Bayes factor based on prior predictive distribution, they also suggest seeking agreement with the other model selection criterion designed to assess local model generalizability (i.e., based on posterior predictive distribution).

The author in [49] describe several interesting points with regards to prior sensitivity. The author views prior sensitivity analysis in theory testing as an opportunity rather than a burden. They argue that it is an attractive feature of a model evaluation measure when psychological models containing quantitatively instantiated theories are sensitive to priors. Ref.  [49] believes that using an informative prior expressing a psychological theory and evaluating models using prior sensitivity measures can serve to advance knowledge. Finally, sensitivity analysis is accessible through an interactive Shiny Application developed by the authors in [50]. The software is designed to help user understand how to assess the substantive impact of prior selection in an interactive way.

## 4. Applications of the Bayes Factor Using R Packages

In this section, we illustrate how to calculate Bayes factors using various techniques available in R, including the R package *BayesFactor* [23]. Various authors have used this package to compute Bayes factors in different settings such as linear correlations, Bayesian *t*-tests, analysis of variance (ANOVA), linear regression, single proportions, and contingency tables [51,52,53,54]. Comparisons between Bayesian and frequentist approaches are provided in the vignettes of [23]. We provide the R code to compute the Bayes factor for a one-sample *t*-test, a multiway ANOVA, a repeated-measures design, and a Poisson generalized linear mixed model (GLM).

### 4.1. One-Sample t-Test

The authors in [52] derived the Jeffreys Zellner Siow (JZS) Bayes factor as a function of the *t*-score and the sample size. To illustrate how the *ttestBF* function of the *BayesFactor* package performs a Bayesian paired *t*-test, they analyzed the *sleep* dataset [55], which includes the variable, i.e., the length of increased sleep (in hours) after taking two drugs when compared to regular nights where no drug was administered. The Bayesian paired *t*-test can evaluate if the levels of effectiveness of two drugs are significantly different (a null hypothesis is that the standardized effect size is zero) [7,52].

Let $y_{1},\ldots ,y_{n}\overset{i.i.d.}{∼}N\left(\sigma \delta ,\sigma^{2}\right)$, where the standardized effect size is given by δ=μ/σ, μ is a grand mean, and $\sigma^{2}$ is the error variance. We test the following hypotheses:

$$H_{0}:\delta =0\;\mathrm{versus}\;H_{1}:\delta \neq 0.$$

The following script of R code implements the Bayesian paired *t*-test and presents the *p*-value of the classical approach for comparison.


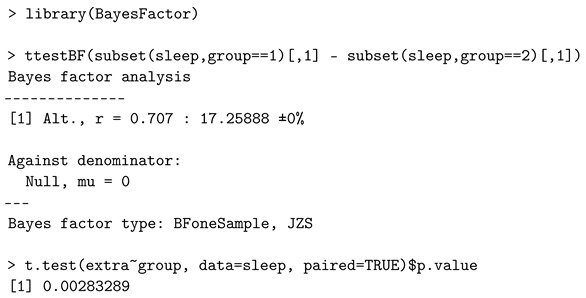


The value $r=0.707\;(\sqrt{2}/2)$ denotes the scale of a Cauchy prior distribution of δ. The Bayes factor value of 17.259 favors the alternative hypothesis, indicating that the effect size is significant in this case. Using the interpretation in Table 1, the evidence against the null hypothesis is “positive”. The classical *p*-value of around 0.3% is also in favor of the alternative, usually considered strong evidence against the null hypothesis.

For this example, the Bayes factor can also be computed by employing a bridge sampling estimate. The R packages *bridgesampling* and *R2jags* used concepts of object-oriented programming and were developed with methods to interact with customizable Markov chain Monte Carlo object routines [41,56]. That is to say, a self-coded in JAGS model can feed the *bridgesampling*’s function *bridge_sampler* to obtain the log marginal likelihood for the model. Their source code (assuming the same priors in [23]) is available at https://osf.io/3yc8q/ (accessed on 28 December 2021). The Bayes factor value in [41] for the *sleep* data is 17.260, which is almost identical to the *BayesFactor* package result, 17.259. Both the *BayesFactor* and *bridgesampling* packages suit the analysis needs. On the one hand, no additional programming knowledge is required to call the functions in the *BayesFactor* package due to the default prior settings, which are user friendly. On the other hand, the *bridgsampling* along with JAGS allows for more sophisticated customization and flexibility in model specifications, which makes more feasible to conduct the sensitivity analysis.

### 4.2. Multiway ANOVA

Consider a two-way ANOVA model $M_{1}:y_{ijk}=\mu +\sigma \left(\tau_{i}+\beta_{j}+\gamma_{ij}\right)+\epsilon_{ijk}$, for i=1,⋯,a, j=1,⋯,b, and k=1,⋯,n, where $y_{ijk}$ is the response for the *k*th subject at the *i*th level of Factor 1 and the *j*th level of Factor 2, μ is the overall mean effect, $\tau_{i}$ is the standardized effect size of the *i*th level of Factor 1, $\beta_{j}$ is the standardized effect size of the *j*th level of Factor 2, $\gamma_{ij}$ is the standardized effect size of the interaction between two factors, $\epsilon_{ijk}$ is a white noise with the mean zero and variance $\sigma^{2}$. We consider comparing the full top-level model $M_{1}$ versus $M_{0}:y_{ijk}=\mu +\epsilon_{ijk}$.

Equivalently, the competing models can be expressed in the matrix-vector form as in [53], i.e.,

$$M_{1}:y=\mu 1+\sigma \left(X_{\tau }\tau +X_{\beta }\beta +X_{\gamma }\gamma \right)+\epsilon \;\mathrm{versus}\;M_{0}:y=\mu 1+\epsilon ,$$

where y is a column vector of *N* observations, 1 is a column vector of *N* ones, τ, β, and γ are column vectors of standardized effect parameters of length *a*, *b*, and ab, respectively, X’s are design matrices, and ϵ ∣ $\sigma^{2}$∼$N(0,\sigma^{2}I)$.

The *anovaBF* function of the *BayesFactor* package compares these linear models (including the reduced models). The *ToothGrowth* dataset [57] is used to study the effects of vitamin C dosage and supplement type on tooth growth in guinea pigs. The *anovaBF* function allows the model comparison (single-factor models, additive model, and full model) against the null model (intercept only). The following script of R code implements the multiway ANOVA.


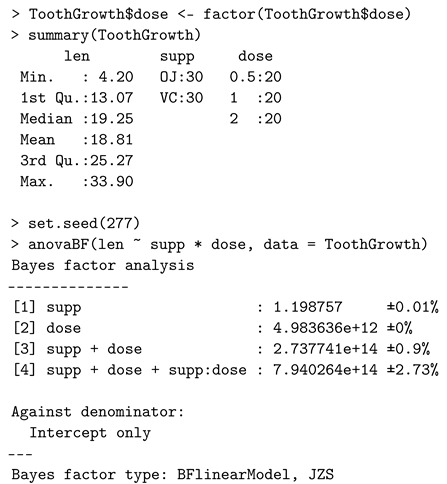


The percentage, e.g., ±2.73% is the proportional Monte Carlo error estimate on the Bayes factor. The Bayes factor value of $7.94\times 10^{14}$ suggests, according to Table 1, very strong evidence in favor of the full model.

It is worth noting that the one-way ANOVA with two levels is consistent with the two-sample *t*-test, when using the default priors. For example, considering the *sleep* data example, one can check that:


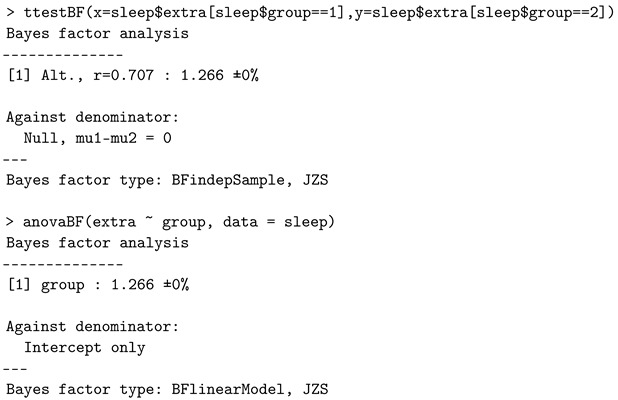


return the same Bayes factor value (but the dataset is not meant for the independent tests).

### 4.3. Repeated-Measures Design

Linear mixed-effects models extend simple linear models to allow both fixed (parameters that do not vary across subjects) and random effects (parameters that are themselves random variables), particularly used when the data are dependent, multilevel, hierarchical, longitudinal, or correlated. In relation to the previous model in Section 4.2, a linear mixed-effects model $M_{1}$ adds the standardized subject-specific random effect $b_{k}$. We now consider comparing

$$M_{1}:y_{ik}=\mu +\sigma \left(b_{k}+\tau_{i}\right)+\epsilon_{ik}\;\mathrm{versus}\;M_{0}:y_{ik}=\mu +\sigma b_{k}+\epsilon_{ik}.$$

We take the *sleep* dataset as an example and specify the argument *whichRandom* in the *anovaBF* function of the *BayesFactor* package, so that it computes the Bayes factor for such a repeated-measures design (or called a within-subjects design). The following script of R code implements the one-way repeated-measures design, where the dataset needs to be in the long format: one column is for the continuous response variable, one column is for the subject indicator, and another column is for the categorical variable indicating the levels.


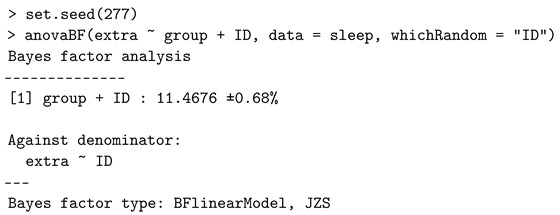


This code generates a Bayes factor of about 11.468 in favor of the alternative hypothesis. The conclusion inferred from the repeated-measures designs is consistent with the earlier paired *t*-test result. One limitation of calling *anovaBF* function is that it only aims to construct the Bayes factor for a homoscedastic case.

### 4.4. Poisson Mixed-Effects Model

A GLM Poisson mixed-effects approach aims to model a discrete count event that was repeatedly measured at several conditions for each subject, e.g., longitudinal studies [58]. The model assumes that the response variable follows a Poisson distribution at the first level. Unlike the cases of normally distributed repeated-measures data, software used to calculate Bayes factors have not been extensively discussed and developed in the context of Bayesian Poisson models. Thus, we illustrate code for sampling the posterior using JAGS, and then the Savage–Dickey density ratio is used to approximate the Bayes factor.

When testing a nested model against an unrestricted alternative, the Bayes factor is computationally and graphically simplified as the ratio calculated by dividing the value of the posterior distribution over the parameters for the alternative model evaluated at the hypothesized value, by the prior for the same model evaluated at the same point [35] and this is the Savage–Dickey density ratio [34]. We demonstrate the use of the Savage–Dickey density ratio described in [59]. We consider fitting a Poisson mixed-effects model to a simulated dataset obtained from Appendix A. We note that the Poisson first level of this example can be changed to many other specifications from the exponential family (e.g., binomial or exponential) with only minor alterations to the code below. With data in the repeated-measures setting, the set of counts obtained from a given subject can be associated. Thus, the standard independence assumption is violated, which is a feature of repeated-measures data.

We utilize the JAGS software and *rjags* R package [60] to fit the model and the *polspline* R package to approximate the log posterior distribution [61] required to evaluate the Savage–Dickey density ratio.


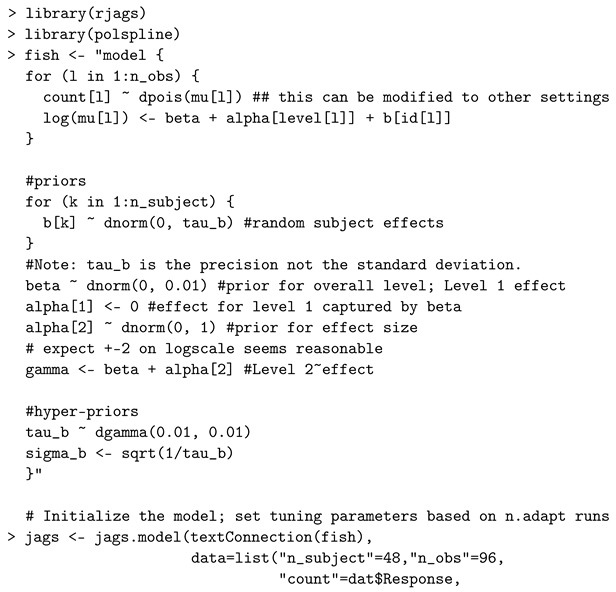



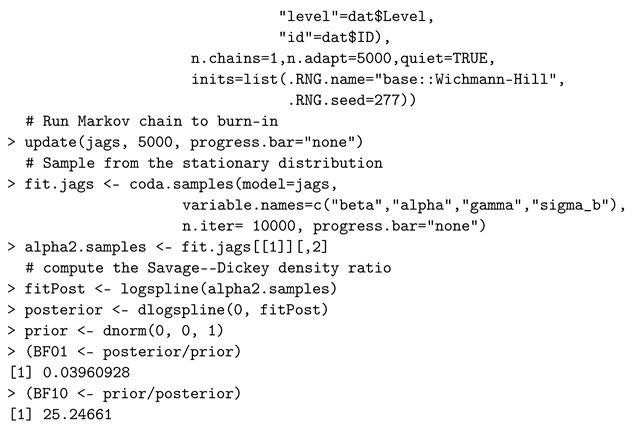


The data are simulated from 48 subjects, and a count is simulated for each of two conditions on each subject. On the log-scale, conditional on the random effects, the mean in condition one is set to $\alpha_{1}=2$ when the data are simulated and the corresponding value is $\alpha_{2}=2.2$ for the second condition. Thus, the data are simulated under the alternative hypothesis. After fitting the model to the simulated data, the Bayes factor in favor of the alternative is $BF_{10}=25.247$ indicating strong evidence in favor of the alternative.

A sensitivity analysis using JAGS or Stan is convenient by passing different parameter values or changing the families of prior distributions. We specified five different prior distributions for the nuisance parameters (the intercept and the precision of the random effects) in the model statement and then examined the Bayes factors computed via the Savage–Dickey density ratio approximation. Four additional combinations of priors are shown in Table 2. Some variation in the value of the Bayes factor is observed though the conclusion remains stable across these prior specifications.

## 5. Summary

We have addressed the activity of hypothesis testing in light of empirical data. Several issues with the classical *p*-values and NHST approaches were reviewed to reach researchers who rarely use Bayesian testing, and NHST is still the dominant vehicle for hypothesis testing. We noted that the debate about the overuse of the *p*-value has been long-lasting, and there are many discussions about the misuse and misinterpretations in the literature.

Following the third principle of the ASA’s statement on *p*-values—i.e., research practice, business, or policy decisions should not solely rely on a *p*-value passing an arbitrary threshold—a Bayesian alternative method based on the Bayes factor was introduced, and the advantages and disadvantages of this approach were brought discussed. One possible caveat of the Bayes factor is its numerical computation, which has been mitigated by the advances of computational resources. We reviewed computational methods employed to approximate the marginal likelihoods, such as the bridge sampling estimator, which has an R package implementation available as an open-source solution.

Issues related to prior distributions were discussed, and we recommended a careful choice of priors via elicitation, combined with prior sensitivity analysis when using Bayes factors as a model selection tool. The Bayesian analysis and hypothesis testing are appealing, but going directly from the NHST to Bayesian hypothesis testing may require a challenging leap. Thus, we showed how, using existing software, one can practically implement statistical techniques related to the discussed Bayesian approach, and provided examples of the usage of packages intended to compute the Bayes factor, namely, in applications of the one-sample *t*-test, multiway ANOVA, repeated-measures designs, and Poisson mixed-effects model.

The Bayes factor is only one of many aspects of Bayesian analysis, and it serves as a bridge to Bayesian inference for researchers interested in testing. The Bayes factor can provide evidence in favor of the null hypothesis and is a relatively intuitive approach for communicating statistical evidence with a meaningful interpretation. The relationships between the Bayes factor and other aspects of the posterior distribution, for example, the overlap of Bayesian highest posterior density intervals, form a topic of interest, and we will report on this issue in another manuscript.

## Figures and Tables

**Table 1:** General-purpose interpretation of Bayes factor values from [17].

${BF}_{10}$	Interpretation of Evidence against $H_{0}$
1 to 3	Not worth more than a bare mention
3 to 20	Positive
20 to 150	Strong
>150	Very Strong

**Table 2:** Prior sensitivity analysis for the Poisson repeated-measures data.

Report	beta	tau_b	${BF}_{01}$	${BF}_{10}$
1	dnorm(0, 0.01)	dgamma(0.01, 0.01)	0.040	25.247
2	dnorm(0, 0.1)	dgamma(0.01, 0.01)	0.054	18.377
3	dnorm(0, 0.01)	dgamma(2, 2)	0.042	24.059
4	dnorm(0, 0.1)	dgamma(2, 2)	0.032	30.859
5	dnorm(0, 0.5)	dgamma(1, 4)	0.023	42.816

## Data Availability

The *sleep* and the *ToothGrowth* datasets are built in R. The Poisson repeated-measures dataset is simulated according to Appendix A.

## Appendix A. Poisson Repeated-Measures Data Simulation

The *sim_Poisson* R function returns a repeated-measures data frame in the long format with 2n rows and three columns. Three columns are subject ID, count response variable, and condition levels.
